# One-year practice effects predict long-term cognitive outcomes in Parkinson’s disease

**DOI:** 10.1177/1877718X251339585

**Published:** 2025-04-29

**Authors:** Sofía Avila Pérez, Vincent Koppelmans, Kevin M Duff, Marit FL Ruitenberg

**Affiliations:** 1 Department of Health, Medical and Neuropsychology, Leiden University, Leiden, the Netherlands; 2 Department of Psychiatry, University of Utah, Salt Lake City, UT, USA; 3 Department of Neurology, Oregon Health & Science University, Portland, OR, USA; 4 Leiden Institute for Brain and Cognition, Leiden, the Netherlands

**Keywords:** cognition, dementia, neuropsychology, parkinson’s disease, practice effects

## Abstract

**Background:**

Predicting which individuals with Parkinson’s disease (PD) will develop cognitive deficits is challenging, but important towards selecting those individuals at higher risk of progression for personalized early intervention and enriching samples for clinical trials of disease modifying agents.

**Objective:**

To examine whether practice effects on cognitive tests across one-year are predictive of eventual cognitive impairment (CI) and dementia (PDD) in individuals with PD.

**Methods:**

Individuals with PD (*n* = 549) from the PPMI database who were cognitively intact at baseline were included for analysis. The Montreal Cognitive Assessment (MoCA) was administered at baseline and during annual follow-up visits over at least five years to determine if participants remained intact (MoCA ≥ 26) or developed CI (MoCA ≤ 25) or dementia (MoCA ≤ 21). Participants also completed a neuropsychological battery at baseline and again after a one-year interval. Practice effects on the cognitive tests across one-year were quantified with standardized regression-based change scores using PPMI data from cognitively intact subjects without PD.

**Results:**

Based on MoCA scores, 39% of patients developed CI and 10% developed PDD during the study. Linear regressions revealed smaller practice effects across one year in people with PD than in controls. Within the PD group, Cox regression analyses showed that smaller practice effects on tests of various cognitive domains were associated with an increased risk for CI. For PDD, only practice effects on a measure of processing speed significantly predicted cognitive outcomes.

**Conclusions:**

These findings demonstrate that practice effects have prognostic value in long-term cognitive outcomes in PD. This has important implications for clinical care and research, as one-year practice effects could help identify individuals at risk for CI and PDD and enrich samples for future clinical trials. Limitations of the present study pertain to the classification of cognitive impairment on the basis of a screening instrument (i.e., the MoCA) without evidence of the absence/presence of functional impairment, and the clinical utility of the one-year interval.

## Plain Language Summary

Although most individuals with Parkinson’s disease (PD) struggle with movement problems (e.g., tremor, slowness), some will also struggle with thinking problems (e.g., paying attention, remembering things). However, it has been difficult to identify which individuals are likely to develop these thinking problems over time, which can limit their access to the most appropriate care. The current study investigated whether practice effects, which are improvements in test scores from taking the same cognitive tests multiple times, could help identify those at risk for problems with thinking. A total of 549 individuals with PD performed various thinking tests at the start of the study and then repeated those same tests one year later. By comparing the difference in performance between these timepoints, practice effects could be measured. Results showed that these practice effects on thinking tests were smaller in individuals with PD than in healthy individuals. Importantly, those individuals with PD who showed smaller practice effects were more likely to experience additional thinking problems over the next five years. The present study is the first to demonstrate that practice effects can help predict future thinking problems in PD. Extending these findings can have important implications for improving clinical care and research in PD.

## Introduction

Parkinson’s disease (PD) is a neurodegenerative disorder characterized by cardinal motor symptoms such as akinesia, bradykinesia, resting tremor, and postural instability. However, many individuals with PD also experience non-motor symptoms, including problems with cognitive functioning. The most frequent cognitive domains affected in PD are memory, attention, executive functions, and visuospatial functions.^1–3^ Approximately 25–27% of individuals with PD have mild cognitive impairment (MCI),^1,4^ and up to 40–50% will develop dementia after 10 years.^5,6^

As the onset and severity of cognitive symptoms vary among individuals with PD, it is difficult to predict if and when an individual will develop cognitive impairment (CI). Aggregation of several neural proteins has been linked to cognitive decline and dementia in PD, including tau and α-synuclein.^1,7,8^ However, quantifying these biomarkers is often expensive and invasive, which can burden participants and deplete resources at centers that serve them. As such, there is need to identify cheaper and non-invasive indicators that predict CI and dementia in PD, which could ultimately select individuals at higher risk of progression for personalized, early intervention and enrichment of samples for clinical trials of disease modifying agents.

Practice effects on neuropsychological tests could prove to be an affordable, non-invasive screening measure for future cognitive outcomes in PD. Practice effects are defined as improvement in cognitive test performance following repeated exposure to test materials.^9,10^ Traditionally, practice effects have been considered a source of error variance when studying data from longitudinal cognitive assessments. However, studies involving clinical populations other than PD indicate that practice effects can be leveraged to serve as a prognostic marker for future cognitive functioning (for a review, see Jutten et al.).^11^ For example, Duff et al.,^9,12^ observed that smaller practice effects on memory measures among patients with MCI portended future cognitive decline. Furthermore, in patients with HIV or Huntington’s disease smaller practice effects on working memory and processing speed measures, respectively, were related to worse, future cognitive functioning.^9^ Hassenstab et al.^13^ showed reduced practice effects on an episodic memory test in cognitively healthy older adults who later progressed to Alzheimer’s disease (AD). However, to our knowledge, the relationship between practice effects and future cognitive functioning has not been explored in PD.

As such, the first aim of the present study was to compare one-year practice effects in individuals with PD to those in healthy controls. It was hypothesized that individuals with PD would exhibit smaller practice effects than healthy controls. Additionally, the study aimed to evaluate if practice effects on neuropsychological tests can predict long-term cognitive outcomes in individuals with PD. To this end, the relationship between one-year practice effects and the progression to CI and PD dementia (PDD) was examined.

## Methods

### Participants

Participants were selected from the Parkinson’s Progression Markers Initiative (PPMI) and data used in the preparation of this article were obtained on 15 May 2023 from the database (https://www.ppmi-info.org/access-data-specimens/download-data), RRID:SCR_006431. For up-to-date information on the study, visit http://www.ppmi-info.org. Independent ethics committees at each clinical site approved the PPMI study and all participants provided written informed consent. The PPMI database includes data from both individuals with PD and healthy individuals who were repeatedly evaluated at three- or six-months intervals over a period of at least five years.

The inclusion criteria for the present study were a PD diagnosis (PD group only), intact cognitive functioning at baseline (see below), and availability of one-year follow-up neuropsychological assessment data (to determine practice effects). Based on these criteria, a total of 547 individuals with PD were selected for this study (223 females; 324 males). They were aged between 29 and 83 years (mean = 61.21 ± 9.95). Additionally, 214 healthy control (HC) subjects (78 females; 136 males), aged between 30 and 83 years (mean = 61.43 ± 11.14) with one-year follow-up neuropsychological assessment data were also included.

### Neuropsychological assessments

Participants completed a neuropsychological test battery at baseline and again after a one-year interval. Table 1 shows the tests that were included in the battery. For most tests, the same version was administered both at baseline and one-year follow-up. However, for the Symbol Digit Modality Test, Hopkins Verbal Learning Test - Revised, and Benton Judgment of Line Orientation test, alternate forms (i.e., different version of the test) were used, which could have resulted in reduced practice effects.

### Cognitive status

Scores on the Montreal Cognitive Assessment (MoCA),^14^ following the recommended education correction of +1 point for ≤12 years of education, were used as a measure of global cognition in order to determine the cognitive status of participants. The MoCA was administered during the screening and at every annual follow-up visit. A MoCA score of ≥26 was used to reflect intact cognition. To classify long-term cognitive outcomes of individuals in our PD group, we used validated, PD-specific MoCA cut-offs to identify subjects who developed CI (score ≤25) and/or PDD (score ≤21).^4,15^ For each individual with PD, this allowed for determining the presence or absence of CI/PDD and the time (in months) until onset thereof.

### Quantifying practice effects

To quantify practice effects, standardized regression-based (SRB) prediction equations were used,^16^ implemented through an in-house developed R-package available to the community through GitHub.^17^ In short, linear regression predicted one-year follow-up scores from baseline scores, age, sex, education, and retest interval using data from HC individuals. The resulting constant and beta weights for each of the predictor variables (one analysis per cognitive outcome) are used to calculate the predicted one-year follow-up scores for each subject from both the HC and PD groups. The difference between the observed one-year follow-up scores and the predicted one-year follow-up scores for each subject are divided by the residual standard deviation of the linear regression model to yield an SRB z-score. The z-scores were trichotomized and compared to a normal distribution, where < −1.645 reflects decline, −1.644–1.644 reflects no change, and > 1.645 reflects improvement between assessment timepoints.^18^ SRB z-scores were reversed for TMT-A and TMT-B, such that all signs went in the same direction with positive values indicating more improvement than expected and negative values indicating less improvement than expected. An overall composite practice effect was determined by averaging SRB z-scores from the LNST, SDMT, SFT, JoLO, HVLT-R immediate recall, and HVLT-R delayed recall.^1^

### Statistical analyses

All analyses were completed with R software version 4.3.0, using the “survival_3.5–7” package. A multiple linear regression analysis with group, age, sex, and education as predictors and SRB z-scores as outcome variable was completed for each of the nine neuropsychological test to evaluate the difference in practice effects between people with PD and HC. Additionally, chi-square analyses were conducted on trichotomized SRB z-scores for each test. This aimed to assess whether the distribution of performance change categories (decline vs. stable vs. improvement) as reflected in the practice effects in the PD group deviated from the expected distribution as observed in the HC group.

Cox proportional hazards models were used to test the hypothesis that smaller one-year practice effects are a risk factor for developing cognitive problems (CI or PDD). Note that these analyses concerned the PD group only. The predictors in these models included age, sex, and SRB z-score, with the outcome measures being the time to onset (in months) of CI and PDD. First, Cox proportional hazards models using the overall composite SRB z-score were examined for CI and for PDD. Furthermore, separate Cox proportional hazards models using SRB z-scores of individual cognitive test scores were run. The proportional hazards assumption was checked by assessing the Schoenfeld’s residuals, which was met for all models. The linearity assumption was not consistently met, with the BNT in the CI models and the JoLO and overall composite in the PDD models showing non-linearity; this was resolved by mean centering the values.

The alpha level threshold for statistical significance was set at 0.05 and multiple comparison correction was applied by running false discovery rate (FDR) correction on the total, single array of *p*-values resulting from both the CI and PDD linear and Cox regression analyses, respectively. To illustrate the link between practice effects and the progression to CI and PDD, we created reversed survival curves; note that for these curves the SRB z-scores were dichotomized into high and low practice effects by means of a median split (this was done for visualization purposes only).

## Results

Of the participants with PD in our sample, 216 individuals (39%) progressed to CI and 53 (10%) to PDD during their participation in the study. The onset of CI ranged from 11–133 months after the initial baseline visit, with a median value of 23 (IQR 13–38). For PDD, the onset ranged from 11–131 months, with a median of 50 (IQR 35–73).

### Comparing practice effects between PD and controls

Results of our regression models are presented in Table 2. The PD group showed smaller-than-expected practice effects compared to the HC group on the LNST, SDMT, TMT-A, TMT-B, and HVLT-R immediate recall (all *β*s< −1.15, all *p*s < 0.026); however, the results for the LNST and TMT-A did not survive FDR correction. Conversely, practice effects were comparable between the PD and HC groups for the BNT, SFT, JoLO, and HVLT-R delayed recall (all *p*s > 0.15).

**Figure 1.**
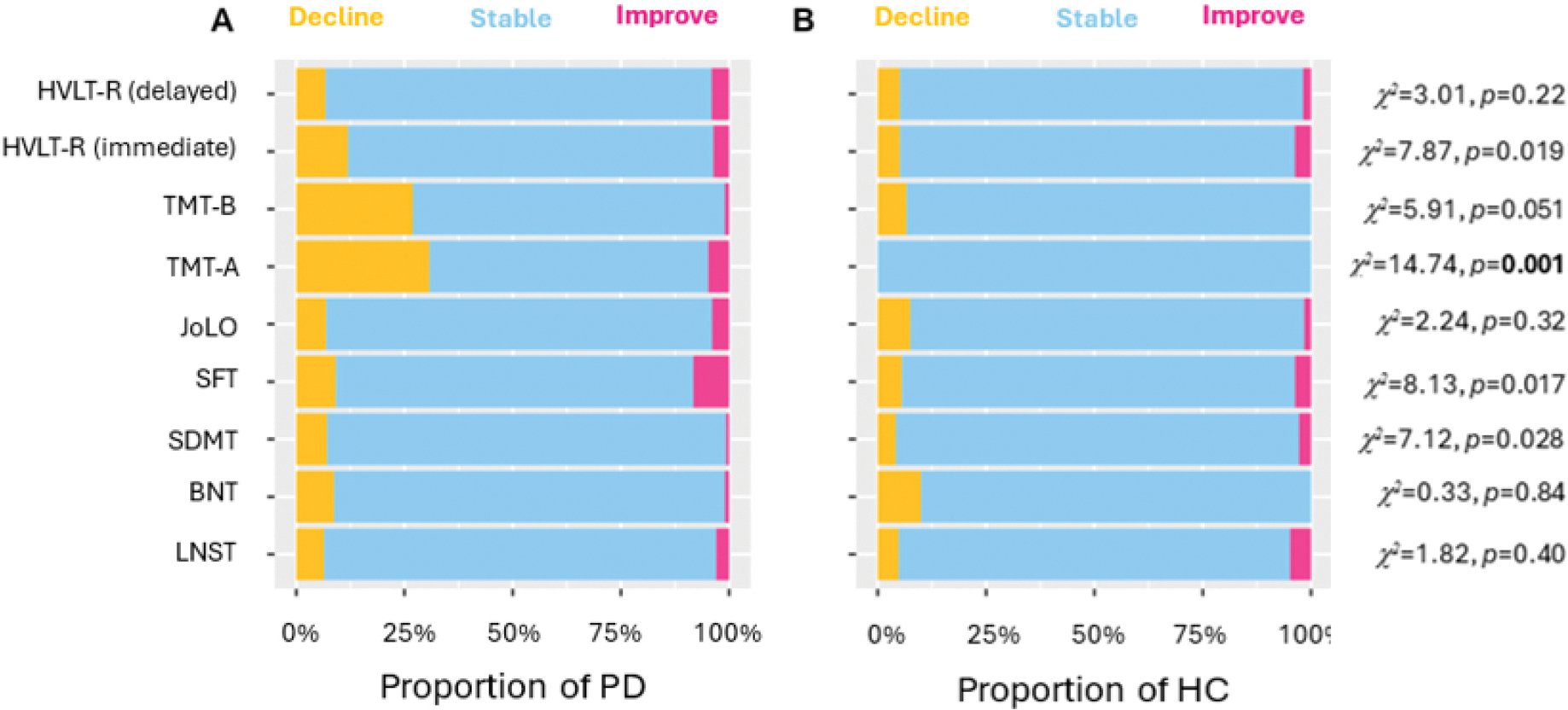
Stacked bar charts showing the distribution of individuals in the PD (A) and HC (B) groups who declined, remained stable, and improved between baseline and 1-year follow-up cognitive tests. Values in bold indicate tests that retain significance after FDR correction.

When SRB z-scores were trichotomized (<−1.645 as decline, −1.644–1.644 as no change, > 1.645 as improvement) and compared to a normal distribution, a greater proportion of individuals from the PD group showed “decline” (or smaller-than-expected practice effects) when compared to the HC group on the SDMT, SFT, TMT-A, and HVLT-R immediate recall (all *χ*^2^s > 7.12, *ps* < 0.028; see Figure 1). However, only the TMT-A finding survived FDR correction. There were no significant distribution differences between PD and HC in any of the other tests (all *p*s > 0.051).

### Practice effects as risk factors for cognitive outcomes in PD

**Figure 2.**
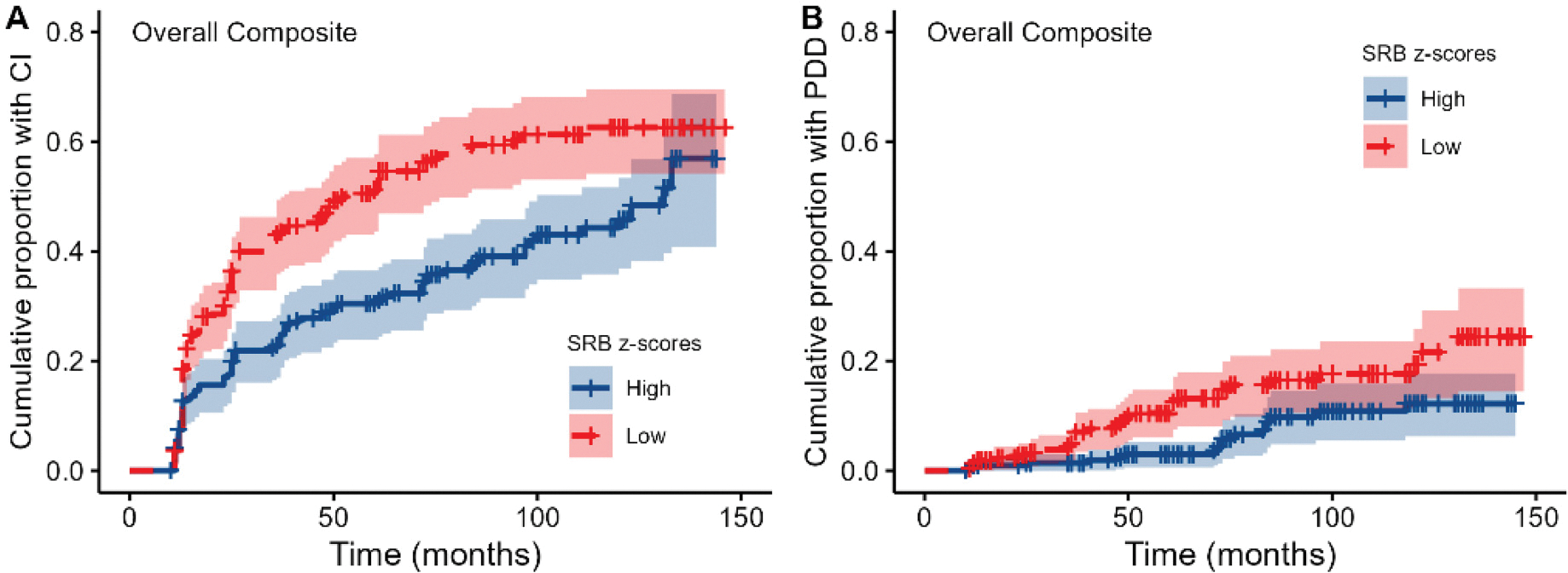
Survival plots showing the cumulative proportion of individuals with PD who developed CI (A) and PDD (B) over time, as predicted by composite SRB z-scores. Note that practice effects were dichotomized into high and low practice effects by means of a median split on the SRB z-scores for visualization purposes only.

As illustrated in Figure 2, results of a Cox proportional hazards model including the overall composite SRB z-score revealed that smaller practice effects were associated with increased risk of developing both CI (HR = 0.46, 95% CI = 0.34–0.60, *p* < 0.001) and PDD (HR = 0.28, 95%CI = 0.14–0.56, *p* = 0.001).

**Figure 3.**
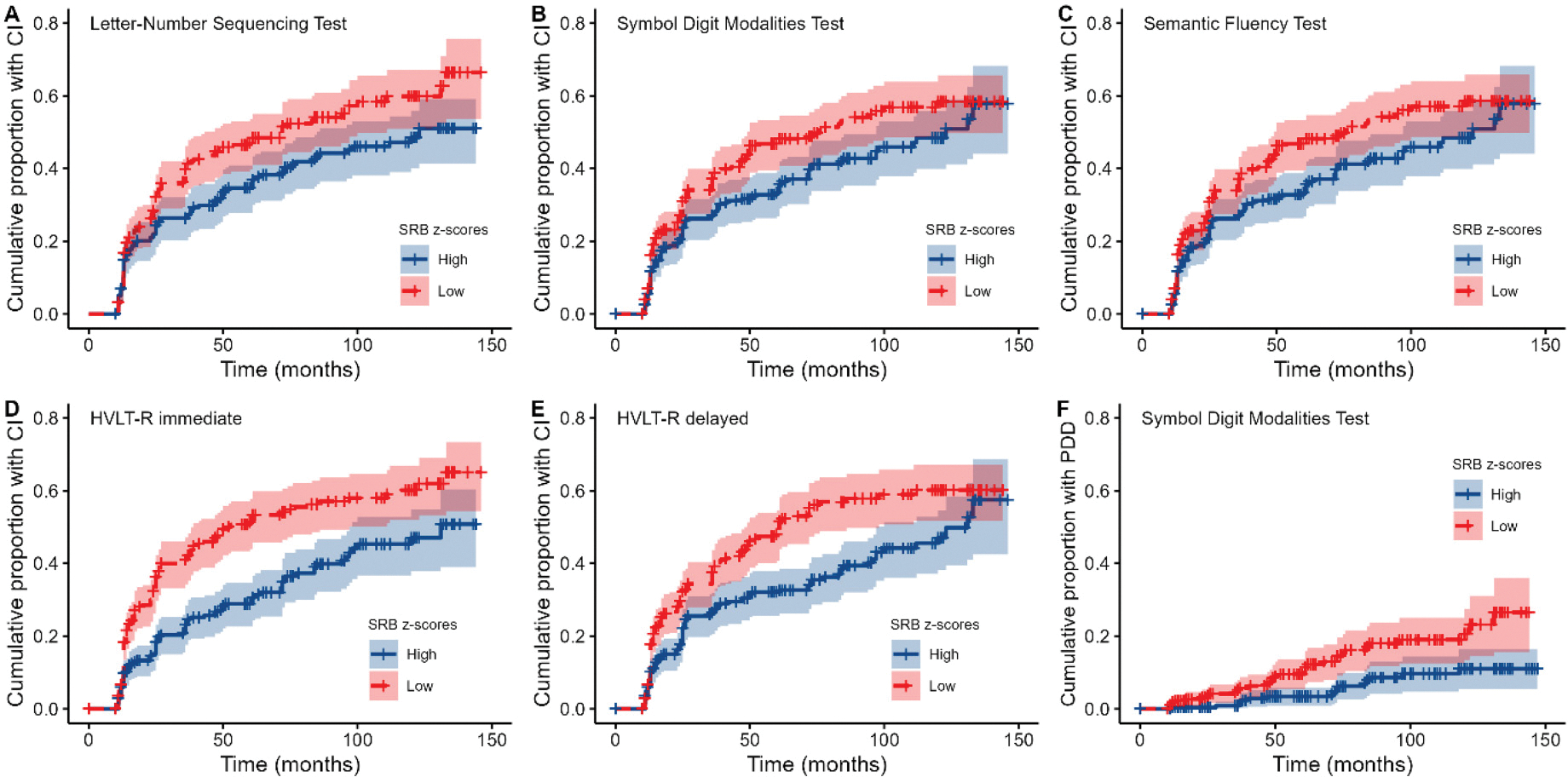
Survival plots showing the cumulative proportion of people with PD who developed CI (A-E) and PDD (F) over time, as predicted by practice effects on individual tests. Note that practice effects were dichotomized into high and low SRB z-scores by means of a median split for visualization purposes only.

When examining SRB z-scores on individual tests, results of Cox proportional hazards models revealed that for various cognitive measures, smaller practice effects were associated with an increased risk of CI at follow-up visits (see Table 3). As illustrated in Figure 3, this concerned the LNST, SDMT, SFT, TMT-B, HVLT-R immediate recall, and HVLT-R delayed recall (all *HR* < 0.85, all *p*s < 0.049). With exception of the TMT-B, all these effects survived FDR correction. For the BNT, JoLO, and TMT-A, practice effects were not associated with future CI (all *p*s > 0.10).

For PDD, results showed that smaller practice effects on the LNST, SDMT, and HVLT-R immediate recall were associated with an increased risk (all *HR* < 0.76, all *p*s < 0.034; see Table 3). However, only the SDMT result survived FDR correction (see Figure 3F). For the SFT, JoLO, and HVLT-R delayed recall, no association between practice effects and PDD was observed (all *p*s > 0.054).

## Discussion

The present study examined whether one-year practice effects on neuropsychological tests differed between individuals with PD and HC. A reduction or absence of practice effects may suggest very subtle cognitive deficits that are not yet detectable through traditional neuropsychological tests. Furthermore, the study explored whether practice effects on neuropsychological tests were predictive of long-term cognitive changes in people with PD. Practice effects and their prognostic value have been examined in Alzheimer’s disease and other neurological conditions, but to our best knowledge have not been considered this way in PD. If practice effects can also predict CI and/or PDD in this population, they could serve as a valuable tool for identifying individuals at higher risk of cognitive decline in PD.

Results of the current study indicate that individuals with PD show smaller-than-expected practice effects across one year on tests assessing attention, processing speed, executive functions, and memory compared to HC. In individuals with PD, across all nine cognitive test scores, their practice effect was approximately one-third of a standard deviation unit smaller than their neurologically intact peers. Perhaps not surprisingly, this reduced practice effect in PD was most notable on tests of processing speed and set shifting (e.g., TMT-A and TMT-B), where the individuals with PD fell nearly one standard deviation unit below the HC subjects. Such results are consistent with prior studies, which have shown impairments in these domains as hallmark features of cognitive impairment in PD.^1–3,19,20^ Through these findings, practice effects might identify very subtle cognitive weaknesses in individuals with PD, before grosser levels of cognitive impairment can be detected with traditional methods. These smaller-than-expected practice effects over one year may capture diminished capacity for adaptation to one’s environment over time, which may lead to the progressive nature of cognitive impairment in PD.

When comparing the distribution of change across the two timepoints between PD and HC, a greater proportion of individuals in the PD group showed decline (or smaller-than-expected practice effects) on measures of processing speed, attention, and verbal fluency. For example, on TMT-A, 31% of the individuals with PD showed decline across one year, where none (0%) of those in the HC group did. This greater proportion of decline on the TMT-A among individuals with PD (which was the only effect surviving correction for multiple comparisons) might potentially be attributed to deterioration of motor rather than cognitive function, as the test has a large motor component. Future work in this area might examine practice effects on purer measures of motor functioning, as a way of separating out the cognitive and motor facets of such tests. Alternatively, future studies could implement measures that minimize the involvement of a motor component, such as the letter–number sequencing task which is an oral version of the TMT that previously has been used and recommended for PD.^19,20^

With regards to the prognostic properties of one-year practice effects, the present results indicate that individuals with PD who display smaller composite practice effects are at increased risk of developing both CI and PDD. As such, clinicians may be able to harness the value of practice effects and preferentially monitor and offer interventions to those individuals with smaller-than-expected practice effects on a neuropsychological test battery. Similarly, researchers might use practice effects as a method of enriching samples in clinical trials on disease-modifying agents. Although composite measures of practice effects may be more reliable, practice effects on specific cognitive tests may be more sensitive in identifying those at most risk for future CI and PDD. When examining specific cognitive domains, it was observed that smaller practice effects on tests of attention, processing speed, executive function, and memory (i.e., LNST, SDMT, SFT, HVLT-R immediate and delayed) were predictive of CI. Furthermore, smaller practice effects on a test of attention and processing speed (i.e., SDMT) were associated with increased risk of PDD. These results, in combination with the literature, suggest that there may be disease-specific cognitive domains for which practice effects are most informative about future cognitive outcomes. For example, Duff et al. (2007)^9^ demonstrated that practice effects on a visuospatial memory test were predictive of cognitive outcomes in individuals with amnestic MCI, while practice effects on a speeded measure of working memory were predictive for HIV positive individuals, and practice effects on a test of attention and processing speed test (i.e., SDMT) were predictive for individuals with Huntington’s disease. The present results in PD, combined with those of Duff et al. (2007)^9^ in Huntington’s disease, suggest that practice effects on tests of attention and processing speed may be particularly sensitive to basal ganglia disorders.

Despite the novel and provocative findings, the current study is not without limitations. First, the current study utilized the MoCA as its measure of CI and PDD instead of clinician ratings or a comprehensive assessment battery. While the MoCA has been shown to provide an accurate cognitive assessment in people with PD,^15^ this instrument is not considered a diagnostic tool but rather a cognitive screener. According to the clinical diagnostic criteria for dementia associated with PD, PDD comprises both cognitive and functional impairment.^21^ However, participants in the present study were classified based on their MoCA scores without taking into account evidence for the absence/presence of functional impairment. We therefore cannot exclude the possibility that for some participants who were classified as PDD, their cognitive difficulties did not affect daily functioning – thus not meeting the diagnostic criteria for PDD. As such, there were likely some false positives and false negatives in the classification of CI or PDD. Additionally, the optimal cut-off scores for establishing cognitive impairment in people with PD based on the MoCA remain a topic of debate. Recently, several studies have used machine learning approaches to evaluate these cut-offs. One study using PPMI data reported that a score of 26 poses the optimal cut-off to discriminate between people with PD and (mild) cognitive impairment versus those without,^22^ which aligns with that used in the present study. However, another study suggests that a MoCA raw score of ≤22 effectively distinguishes people with PD and (mild) cognitive impairment from those with normal cognition,^23^ which is lower than the cut-off of ≤25 used in the present study. Future studies should adopt a more comprehensive clinical evaluation including both cognitive and functional assessments to more reliably determine cognitive status.

A second limitation is that predictive models for PDD were not possible for two neuropsychological tests (i.e., the BNT and TMT) due to the small number of subjects with these tests. This may pertain to PDD typically taking longer to develop—the time from PD disease onset to PDD is approximately 10–15 years,^5,24^ which is considerably longer than the follow-up time in the present study. Furthermore, the relatively low proportion of PDD in the current sample may have impacted the possibility of detecting significant associations with most cognitive variables. It therefore remains unclear to what extent practice effects on these specific tests (or other cognitive abilities that were not assessed in the PPMI study [e.g., construction, visual memory]) are associated with the risk of developing PDD. Finally, although the current study provides some proof of concept, examining practice effects over a one-year test-retest interval will likely be too long for clinical decision-making or clinical trials methodology. In Alzheimer’s disease, future cognitive functioning can be predicted based on practice effects with intervals as brief as 1 week (for a review, see Jutten et al.).^11^ As such, cognitive changes in PD should be examined over shorter time frames. Future research might also consider more frequent assessments and investigating the prognostic value of practice effects in other conditions with cognitive and motor impairments (e.g., dementia with Lewy bodies).

Despite these limitations, the present findings underscore the prognostic value of one-year practice effects in identifying people with PD at risk of developing CI or PDD. Practice effects could serve as an inexpensive, widely-accessible, and non-invasive marker for monitoring an individual’s cognitive status. This could help clinicians in making decisions about tailored treatments, as practice effects could serve as a screening tool for identifying those in need of early intervention. Furthermore, practice effects might be useful to select participants for clinical trials, for example aimed at prevention or cognitive rehabilitation.

## Figures and Tables

**Table 1.** Overview of cognitive tests administered, including the number of participants, brief test descriptions, and task measures.

Cognitive test	Participants	Task description	Measure

WMS-III Letter-Number Sequencing Test (LNST)	PD: 435 HC: 185	Verbally reporting back an auditory series of letters and digits	Number of correctly recalled sequences (score range 0–21)
Boston Naming Test (BNT)	PD: 103 HC: 30	Naming objects from visually presented line drawings	Number of spontaneously given correct responses (score range 0–60)
Symbol Digit Modalities Test (SDMT) *	PD: 547 HC: 214	Writing symbols that are paired with numbers using a key	Number of correct responses in 90 s (score range 0–110)
Semantic Fluency Test (SFT)	PD: 546 HC: 214	Generating words from a given category	Number of animals named in 60 s
Benton Judgment of Line Orientation (JoLO) *	PD: 542 HC: 185	Matching the angle and orientation of lines in space	Number of correct answers (score range 0–15)
Trail Making Test Part A (TMT-A)	PD: 104 HC: 30	Connecting 24 randomly positioned numbers in numeric order	Completion time (sec)
Trail Making Test Part B (TMT-B)	PD: 104 HC: 30	Connecting randomly positioned numbers and letters in order	Complete time (sec)
Hopkins Verbal Learning Test - Revised Immediate Recall (HVLT-R immediate) *	PD: 546 HC: 214	Recalling of series of 12 verbally presented words over three trials	Number of correctly recalled words (score range: 0–12 per trial)
Hopkins Verbal Learning Test - Revised Delayed Recall (HVLT-R delayed) *	PD: 546 HC: 214	Recalling the series of 12 verbally presented words after a 20–25 min delay	Number of correctly recalled words (score range 0–12)

* indicates tests for which alternate forms were used.

**Table 2.** Raw test scores at baseline and one-year neuropsychological assessments and practice effects (SRB z-scores) for the PD and HC groups (all M ± SD). The right column shows the results of the linear regressions comparing SRB z-scores between PD and HC.

Test	PD			HC			Group difference
	Baseline score	1 -year score	SRB z-score	Baseline score	1 -year score	SRB z-score ^a^	

LNST	10.81 ± 2.65	10.49 ± 2.65	−0.20 ± 0.97	10.85 ± 2.59	10.90 ± 2.66	0.00 ± 0.98	*β*=−0.20, *p* = 0.021
BNT	54.09 ± 10.88	55.69 ± 8.81	0.14 ± 0.97	55.46 ± 8.14	55.06 ± 9.56	0.00 ± 0.90	*β*=0.11, *p* = 0.55
SDMT	42.36 ± 9.96	42.10 ± 10.08	−0.36 ± 0.87	47.10 ± 10.26	47.62 ± 10.55	−0.00 ± 0.99	*β*=−0.36, p **< 0.001**
SFT	21.84 ± 5.34	21.96 ± 5.44	−0.13 ± 1.17	22.16 ± 5.28	22.66 ± 5.19	0.00 ± 0.99	*β*=−0.I3, *p* = 0.15
JoLO	12.77 ± 2.17	12.37 ± 2.37	0.01 ± 1.02	13.08 ± 2.01	12.65 ± 2.45	−0.00 ± 0.98	*β*=−0.00, *p* = 0.99
TMT-A	34.57 ± 10.92	34.08 ± 13.82	−0.89 ± 2.30	28.4 ± 10.27	28.13 ± 5.65	−0.00 ± 0.90	*β*=−0.95, *p* = 0.026
TMT-B	73.03 ± 26.79	77.06 ± 32.64	− 1.05 ± 2.13	56.86 ± 20.53	59.9 ± 15.68	−0.00 ± 0.90	*β*=−1.15, *p* = **0.003**
HVLT-R (immediate)	25.37 ± 4.78	24.76 ± 5.31	−0.24 ± 1.13	26.25 ± 4.56	26.14 ± 4.70	0.00 ± 0.99	*β*=−0.25, *p* = **0.004**
HVLT-R (delayed)	8.86 ± 2.44	8.73 ± 2.65	−0.03 ± 1.13	9.33 ± 2.34	9.08 ± 2.47	−0.00 ± 0.99	*β*=−0.04, *p* = 0.63

Values in bold indicate tests that retain significance after FDR correction.

a SRB z-scores are rounded to two decimals; exact values ranged from −4.23e-15 to 5.46e-17.

**Table 3.** Results from the Cox proportional hazards models for CI and PDD as a function of neuropsychological tests.

Test	Cox model for CI			Cox model for PDD		
	HR	95% CI	*p*	HR	95% CI	*p*

LNST	0.78	0.66–0.91	**0.001**	0.69	0.49–0.97	0.034
BNT	0.77	0.42–1.41	0.40	n/a	n/a	n/a
SDMT	0.82	0.70–0.96	**0.019**	0.54	0.39–0.75	**0.001**
SFT	0.85	0.75–0.95	**0.004**	0.78	0.61–1.00	0.057
JoLO	0.89	0.77–1.02	0.10	0.82	0.58–1.18	0.29
TMT-A	0.86	0.67–1.09	0.22	n/a	n/a	n/a
TMT-B	0.80	0.64–0.99	0.049	n/a	n/a	n/a
HVLT-R (immediate)	0.85	0.75–0.97	**0.016**	0.76	0.60–0.97	0.027
HVLT-R (delayed)	0.85	0.75–0.96	**0.013**	0.79	0.63–1.00	0.054

95% CI: 95% confidence interval. Values in bold indicate tests that retain significance after FDR correction. Models for the BNTand TMT measures could not be developed for PDD, because none of the participants in the small sample that completed these tests developed PDD.

## Data Availability

This study involves data acquired from the PPMI (http://www.ppmi-info.org).
